# A study on immunomodulatory mechanism of Polysaccharopeptide mediated by TLR4 signaling pathway

**DOI:** 10.1186/s12865-015-0100-5

**Published:** 2015-06-02

**Authors:** Zhixue Wang, Bing Dong, Zifang Feng, Shuang Yu, Yixi Bao

**Affiliations:** Department of Clinical Laboratory, the Second Affiliated Hospital of Chongqing Medical University, Lin Jiang Rd. #76, Yuzhong District, 400010 Chongqing, China

**Keywords:** Polysaccharopeptide, TLR4, TLR4 signaling pathway, Immunomodulatory

## Abstract

**Background:**

Polysaccharopeptide (PSP), isolated from *Coriolus versicolor* COV-1 strain, is a protein-bound polysaccharide widely used as immunoadjuvant for cancer immunotherapy. Although the immunomodulatory activity of PSP has been well established, the precise molecule mechanisms of its biological activity have yet to be fully elucidated.

**Methods:**

In the present study, we first investigated the immunomodulatory activity of PSP in peritoneal macrophages from C57BL/10J (TLR4^+/+^) and C57BL/10ScCr (TLR4^-/-^) mice carrying a defective toll-like receptor-4 (TLR4) gene and then evaluated PSP for its effect on tumor inhibition rates and the immune organ index in above two different strains of mice. In addition, PSP were also evaluated for its activation of TLR4, TLR4-downstream molecules (TRAF6, NF-κB and AP-1) in spleens of tumor-bearing C57BL/10J (TLR4^+/+^) and C57BL/10ScCr (TLR4^-/-^) mice.

**Results:**

The results showed that PSP had adjuvant activities in stimulating expressions of cytokines as well as TLR4, TRAF6, phosphorylation of NF-κB p65 transcription factors and phosphorylation of c-Jun (a component of the transcription factor AP-1) in peritoneal macrophages from C57BL/10J (TLR4^+/+^) mice but not from C57BL/10ScCr (TLR4^-/-^) mice. In vivo PSP as well as Adriamycin (ADM) decreased the mean weights of tumors compared with normal saline and PSP increased thymus index and spleen index relative to ADM in tumor-bearing C57BL/10J (TLR4^+/+^) mice but not in C57BL/10ScCr (TLR4^-/-^) mice.

**Conclusions:**

We demonstrated that PSP activates peritoneal macrophages in vitro via TLR4 signaling pathway and PSP functions its immunoregulatory effect in vivo also via TLR4 signaling pathway. These data strongly suggest TLR4 signaling pathway is involved in PSP-mediated immunomodulatory activities.

## Background

Historically, Mushrooms have been considered as important source of materials in traditional Chinese medicine. Polysaccharide extracts from mushrooms display immunomodulatory and anti-tumor activities in vivo and in vitro [1–3]. *Coriolus versicolor* (better known as Yunzhi in China), a medicinal fungus of the Basidiomycetes family, has been used as a “magic herb” for promoting good health and longevity. Its medicinal value was recorded in the *Compendium of Materia Medica* and *Shen Non Compendium Medica* thousands of years ago in China [4–6]. Polysaccharopeptide (PSP) is a protein-bound polysaccharide extracted from the deep-layer cultivated mycelia of C. versicolor COV-1 strain [7]. PSP, which has an approximate molecular weight of 100KDa and is highly water-soluble, appears to be safe during pregnancy. It did not affect ovarian steroidogenesis, ovulation and midterm gestation in mice [4, 8, 9]. Numerous scientific investigations have demonstrated that PSP has anti-tumor, anti-inflammatory, immunoregulatory and antiviral effects in vivo and in vitro [5, 10–12]. PSP was also found to restore a depressed immunological responsiveness in patients suffering from cancer or in chemotherapy [13, 14]. However, the underlying molecular mechanisms involved in those functions have not been clearly elucidated.

Recent researches indicated that the immunoregulatory effects of polysaccharides are related to the Toll-like receptors (TLR) signaling pathway [15, 16], and Toll-like receptors 4 (TLR4) plays a central role in the enhancement of the innate immune response and the production of cytokine induced by polysaccharides [17–20]. A family of TLRs plays an important role in the recognition of molecular structures that are shared by many pathogens in the host defense system [21, 22]. TLR4 is the first mammalian homologue of the Drosophila Toll protein [23] and recognizes lipopolysaccharide (LPS) from Gram-negative bacteria, which causes septic shock [24, 25]. Further study indicates that TLR4 is the immune receptor of both Ganoderma lucidum polysaccharides (GLPS) and polysaccharides from Astragalus membranaceus [26, 27]. In addition, our previous studies have reported that PSP has an immunoregulatory effect through the TLR4 signaling pathway in human peripheral blood mononuclear cells (PBMCs) [28].

To further elucidate the molecular mechanisms for the immunoregulatory function of PSP, based on previous evidences, we focused on investigating the role of TLR4 and TLR4 signaling pathway in the PSP-mediated immunomodulation activities. In this paper, C57BL/10ScCr mice (TLR4-defect mice lacking functional TLR4), C57BL/10 J mice (wild-type mice with functional TLR4) and the peritoneal macrophages isolated from these two strains were used to demonstrated the immunomodulation mechanism of PSP mediated by TLR4 signaling pathway both in vitro and in vivo.

## Results

### PSP-induced activation of macrophages through TLR4

**Fig. 1:**
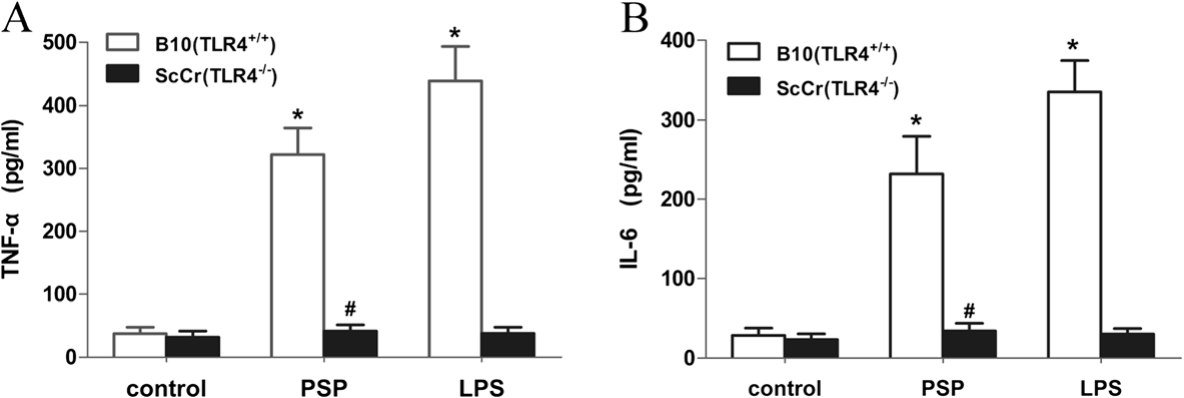
PSP-induced TNF-α and IL-6 production by mouse peritoneal macrophages. B10 (TLR4^+/+^) and ScCr (TLR4^−/−^) mouse peritoneal macrophages were treated with PSP (25 μg/ml) or LPS (100 ng/ml) for 24 h. TNF-α (**a**) or IL-6 (**b**) concentration (pg/ml) in the culture supernatant was determined by ELISA. Data represent the mean ± SD of three experiments. *p < 0.05 vs. control (B10). #*p* < 0.05 vs. PSP (B10)

The activation of macrophages facilitates the production of many immunomodulatory substances including cytokines. In order to verify if TLR4 was required for PSP activation of macrophages, peritoneal macrophages from ScCr (TLR4^−/−^) and B10 (TLR4^+/+^) mice were incubated with PSP (25 μg/ml) or LPS (100 ng/ml) as a positive control for 24 h and then assayed for TNF-α and IL-6 concentration in their culture supernatant. As shown in Fig. 1, both LPS and PSP induced TNF-α secretion by peritoneal macrophages from wild-type control B10 mice but did not induce TNF-α secretion in ScCr mice lacking functional TLR4 (Fig. 1a). Similarly, LPS and PSP failed to induce IL-6 secretion in ScCr mice (Fig. 1b). These results suggest that TLR4 is involved in PSP activation of murine macrophages.

### Effect of PSP on activation of TLR4 signaling pathway in peritoneal macrophages from B10 (TLR4^+/+^) and ScCr (TLR4^−/−^) mice

**Fig. 2:**
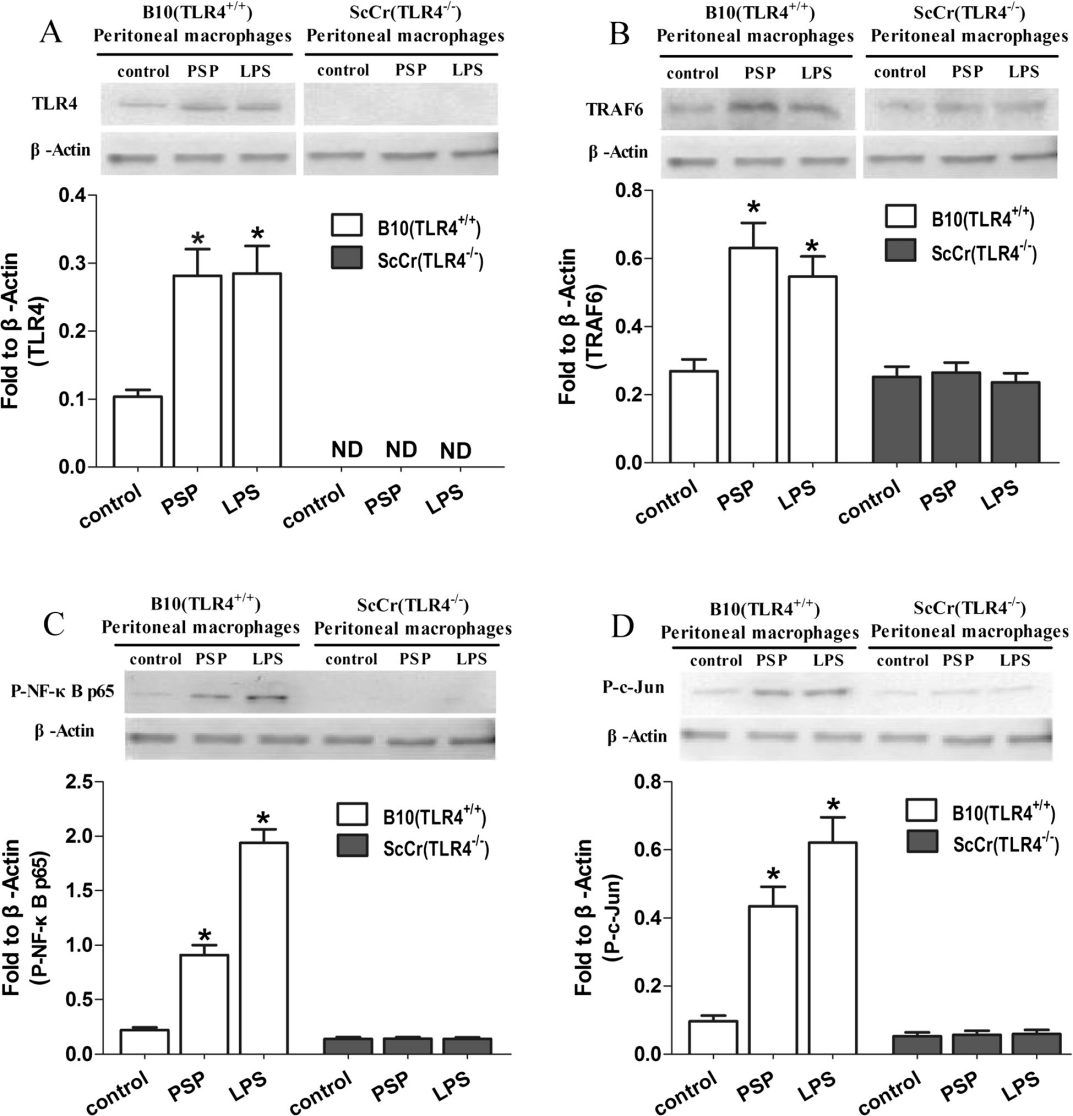
PSP-mediated activation of TLR4, TRAF6, NF-κB and AP-1 in B10 (TLR4^+/+^) and ScCr (TLR4^−/−^) peritoneal macrophages. Peritoneal macrophages from B10 (TLR4^+/+^) and ScCr (TLR4^−/−^) mice were cultured with PSP (25 μg/ml) or LPS (100 ng/ml) for 12 h. The levels of TLR4 (**a**), TRAF6 (**b**), P- NF-κB p65 (**c**) and P-c-Jun (**d**) were examined by Western blot. Data represent the mean ± SD of three experiments. *p < 0.05 vs. control group. ND = not detected

TLR4 activates intracellular signaling pathways that lead to the induction of cytokines, such as TNF-α and IL-6 [29, 30]. To elucidate TLR4 signaling pathway involved in PSP-mediated macrophage activation, we examined activation of TLR4, TLR4-downstream molecules (TRAF6, NF-κB and AP-1) in TLR4^+/+^ and TLR4^−/−^ peritoneal macrophages at protein level. After incubation with PSP (25 μg/ml) or LPS (100 ng/ml) for 12 h in 5 % CO_2_ at 37 °C, western blot was performed. As shown in Fig. 2, the protein levels of TLR4 and TRAF6 were upregulated in PSP and LPS group compared with control group in peritoneal macrophages of B10 (TLR4^+/+^) mice (all *p* < 0.05). The phosphorylation levels of transcription factors NF-κB (p65 subunit) and AP-1 (P-c-Jun) were likewise significantly higher in PSP and LPS group than control group in peritoneal macrophages of B10 (TLR4^+/+^) mice (all *p* < 0.05). No significant differences in those protein expressions were found between groups in ScCr (TLR4^−/−^) mice, suggesting that the activation was dependent on TLR4 signaling pathway.

### General state of tumor-bearing mice

After administration, there was no abnormality seen daily in the autonomic activities, behavior, ingestion, pelage, feces, and urine of mice in each group. Also, there were no abnormal secretion in the eye, ear, nose, and mouth.

### Effect of PSP on tumor inhibition rates and the immune organ index in B10 (TLR4^+/+^) and ScCr (TLR4^−/−^) tumor-bearing mice

**Fig. 3:**
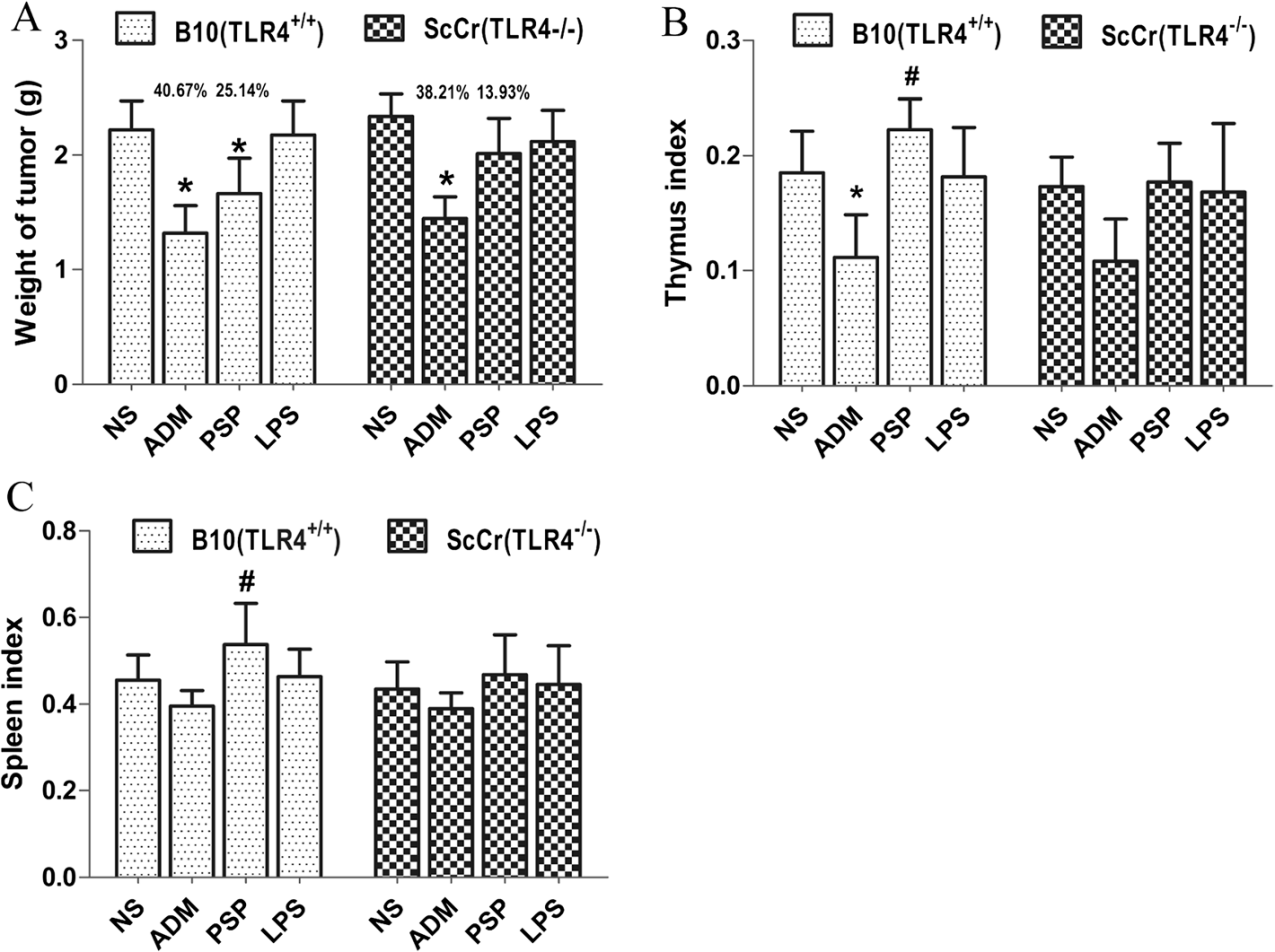
The tumor weight (g) and the tumor inhibition rate (%) of each group (**a**); the indexes of immune organs in each group: The thymus index of each group (**b**), The spleen index of each group (**c**). Data represent the mean ± SD; n = 4-6 mice/ group. *p < 0.05 vs. saline group; #*p* < 0.05 vs. ADM group

In vivo immunoregulatory and anti-tumor activities of PSP were examined in B10 (TLR4^+/+^) or ScCr (TLR4^−/−^) mice inoculated with Ehrlich’s ascites carcinoma (EAC) cells. Twenty five days after inoculation, the mean weights of tumors in B10 (TLR4^+/+^) tumor-bearing mice that received PSP and ADM treatment were significantly decreased compared with those in the saline group (all *p* < 0.05). The inhibition rates of ADM group and PSP group were 40.67 % and 29.64 %. In ScCr (TLR4^−/−^) mice, the mean weights of tumors in the ADM group were decreased significantly compared with those in the saline group (*p* < 0.05),however, there were no significant change trends between PSP group and saline group (Fig. 3a). Furthermore, in B10 (TLR4^+/+^) tumor-bearing mice, the thymus index in the ADM group decreased significantly, as compared with that in the saline group (*p* < 0.05). Both the thymus index and spleen index in the PSP group increased significantly relative to that in the ADM group (all *p* < 0.05). However, there were no significant differences of the thymus index and spleen index among groups in ScCr (TLR4^−/−^) mice (Fig. 3b and c).

### TLR4 expression and signaling in spleens of B10 (TLR4^+/+^) and ScCr (TLR4^−/−^) mice exposed to PSP

**Fig. 4:**
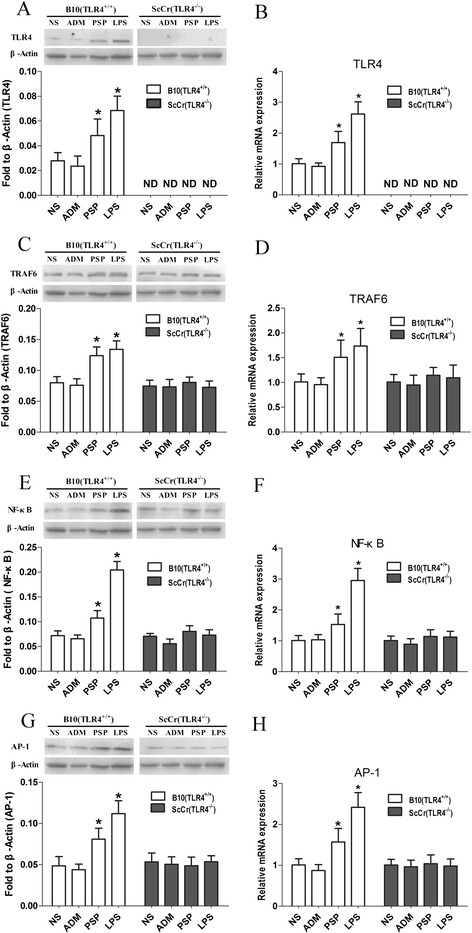
PSP-induced changes in TLR4, TRAF6, NF-κB and AP-1 expressions in spleens of B10 (TLR4^+/+^) and ScCr (TLR4^−/−^) tumor-bearing mice. The mRNA level of TLR4 (**b**), TRAF6 (**d**), NF-κB (**f**) and AP-1 **(h)** in spleens of B10 (TLR4^+/+^) and ScCr (TLR4^−/−^) mice were measured by Q-PCR. The protein level of TLR4 (**a**), TRAF6 (**c**), NF-κB (**e**) and AP-1 (**g**) were tested by Western blot. Data represent the mean ± SD; n = 4-6 mice/ group. *p < 0.05 vs. saline group. ND = not detected

The signaling pathway mediated by TLR4 has been implicated in the response to PSP in vitro. To clarify whether TLR4 signaling pathway is involved in PSP-mediated immunomodulation in vivo, we studied the expression at mRNA and protein level of TLR4, TRAF6, NF-κB and AP-1 in the spleen of B10 (TLR4^+/+^) and ScCr (TLR4^−/−^) mice. Twenty five days after administration, TLR4, TRAF6, NF-κB and AP-1 in PSP and LPS group (positive control) were significantly increased at mRNA and protein level relative to those of the saline group in the spleen of B10 (TLR4^+/+^) tumor-bearing mice (all *p* < 0.05). As expected, there were no significant differences in the expressions of TLR4, TRAF6, NF-κB and AP-1 among groups in ScCr (TLR4^−/−^) mice (Fig. 4).

## Discussion

PSP is well-known for its immunoregulatory effect, attaining widespread usage as therapeutic adjuvant for cancer immunotherapy in China and Japan [4]. Previous studies have indicated that PSP could significantly increase the percentage of CD4^+^ T lymphocytes, the ratio of CD4^+^/CD8^+^ and the quantity and percentage of the B lymphocytes and finally enhanced the immune system of cancer patients [14, 31]. However, studies concerning the underlying mechanisms involved in PSP-mediated immunomodulation effects are very limited. Our previous study demonstrated that PSP has an immunoregulatory effect through the TLR4 signaling pathway in PBMCs from breast cancer patients [28].

The role of TLR4 in Gram-negative bacterial LPS-mediated signaling has been studied extensively [32, 33]. Our current results indicated that both PSP and LPS transduces part of signalings via the TLR4, but the biological outcomes are quite different. Interestingly, PSP has been used to promote health in many Asian countries, but concentrated LPS of contaminated bacteria causes high mortality in vitro or in vivo [19]. However, the molecular mechanisms for PSP or LPS in the differential immunity outcomes are not clear.

In our study, to delineate the signaling pathway involved in PSP-mediated immunomodulation in vitro, peritoneal macrophages from ScCr (TLR4^−/−^) and B10 (TLR4^+/+^) mice were used. Macrophages, which provide an important bridge between innate and adaptive immunity, play critical roles in host defense, including phagocytosis of pathogens and apoptotic cells, production of cytokines, and proteolytic processing and presentation of foreign antigens [34, 35]. The activation of macrophages facilitates the production of many immunomodulatory substances including cytokines, such as TNF-α, IL-6 and IFN-γ, which are known to play an important role in suppressing tumor cells. Indeed, a variety of plant polysaccharides have been shown to possess immunomodulatory activity through their ability to modulate macrophage function [36]. When incubated with PSP (25 μg/ml) and LPS (100 ng/ml) for 24 h, peritoneal macrophages from B10 (TLR4^+/+^) mice increased their production of TNF-α and IL-6. However, production of TNF-α and IL-6 in PSP and LPS stimulated peritoneal macrophages from ScCr (TLR4^−/−^) was significantly reduced (Fig. 1). Moreover, PSP and LPS upregulated the expressions of TLR4 and TLR4-downstream molecules (TRAF6, Phospho-NF-κB p65 and Phospho-c-Jun) in B10 (TLR4^+/+^) peritoneal macrophages but not in ScCr (TLR4^−/−^) peritoneal macrophages (Fig. 2). As TLR4 recognizes LPS from most Gram-negative species, LPS was used as the prototypical TLR4 agonist to provide a positive reference. These results suggest that TLR4 signaling pathway is involved in PSP-induced activation of macrophages in vitro.

To verify whether TLR4 signaling pathway is involved in PSP-mediated immunomodulation in vivo, we found PSP and ADM treatment significantly decreased the mean weights of tumors than saline treatment in B10 (TLR4^+/+^) tumor-bearing mice (Fig. 3a). At the same time, PSP administration significantly increased the thymus index and spleen index relative to ADM administration in B10 (TLR4^+/+^) mice (Fig. 3b and c). However, these significant changes were not observed in ScCr (TLR4^−/−^) tumor-bearing mice (Fig. 3). These results indicate that PSP could inhibit tumor growth and alleviate the decrease of thymus and spleen induced by ADM via TLR4 signaling pathway.

In vivo, the activation of TLR4 and TLR4-downstream molecules (TRAF6, NF-κB and AP-1) in spleens of B10 (TLR4^+/+^) and ScCr (TLR4^−/−^) tumor-bearing mice were measured by Q-PCR and Western blot. In Q-PCR experiments, Vandesompele method, which is more advanced than the Pfaffl method, was applied to analyze these data. The Vandesompele method normalizes not only with the adjusted PCR efficiency but also with multiple genes. β-actin and GAPDH were used as reference genes to assay the relative mRNA expression levels. LPS was used as positive reference like in vitro study. From the results, we found that the mRNA and protein levels of TLR4, TRAF6, NF-κB and AP-1 were significantly upregulated by PSP as well as LPS in spleens of B10 (TLR4^+/+^) tumor-bearing mice (Fig. 4). At the same time, these significantly differences were not observed in ScCr (TLR4^−/−^) mice (Fig. 4).

## Conclusions

In summary, our present study demonstrated that PSP activates peritoneal macrophages in vitro via TLR4 signaling pathway and PSP functions its immunoregulatory effect in vivo also via TLR4 signaling pathway. These data strongly suggest TLR4 signaling pathway is involved in PSP-mediated immunomodulatory activities.

## Methods

### Animals

Female 4–6 weeks old C57BL/10ScCr (ScCr, TLR4^−/−^) and C57BL/10 J (B10, TLR4^+/+^) mice (Permit number: scxk 2010–0001), weighing 16-20 g, were purchased from Model Animal Research Center of Nanjing University (Nanjing, China). The C57BL/10ScCr murine strain has a homozygous deletion of 74,723 bp at the *tlr4* locus, removing all three exons, which abolishes the response to LPS in these mice [24]. The C57BL/10 J murine strain was used as the wild-type control group. Both strains will be described as ScCr and B10 throughout the article. All experiments were followed the protocols approved by the Animal Care and Use Committee of the Second Affiliated Hospital of Chongqing Medical University.

### Preparation of PSP solution

PSP, isolated from Coriolus versisicolor COV-1, was obtained from Jiangsu shenhua pharmaceutical Co., LTD (Jiangsu, China). It was characterized as previously described [28]. Gram-negative bacterial endotoxin level of the PSP was measured by using chromogenic end-point tachypleus amebocyte lysate (CE TAL) assay kit (Chinese Horseshoe Crab Reagent Manufactory Co., Ltd., Xiamen, China) according to the manufacturer’s instruction in our previous study, the results showed that very low endotoxin level (below 0.34 EU/ml) was found in PSP at 100 μg/ml, indicating that endotoxin contamination in PSP was negligible [28]. In our study, PSP was dissolved in physiological saline for in vivo experiments or in plain RPMI-1640 medium (HyClone, Thermo scientific, USA), sterilized by passing through a 0.22-μm filter (Millipore, USA) and stored at −20 °C for future use.

### Preparation of tumor cells

Ehrlich’s ascites carcinoma (EAC) cells were purchased from Nanjing KeyGEN biotech Co., Ltd. (Nanjing, China), cultured and passaged in abdominal cavity of mice.

### Isolation of mouse peritoneal macrophages

Peritoneal macrophages were obtained from ScCr and B10 mice by intraperitoneal injection of sterile thiolglycollate medium. Four days after injection peritoneal macrophages were harvested from mice.

### Modeling of the tumor-bearing mice

Murine EAC cells were cultured in the abdominal cavity of mice for 7–8 days. They were taken out from the ascites, diluted to the concentration of 1 × 10^7^cells ml^−1^ with sterilized physiological saline. Then 0.1 mL EAC cell suspension was inoculated subcutaneously to the right armpit of each mouse. Generally, the solid tumor could be palpated after 7–10 days with an achievement ratio of nearly 100 %. After inoculation, each mouse was weighed immediately. After 24 h, intraperitoneal injection and intragastric administration were carried out simultaneously.

### Experimental design

For in vitro experiments, the cultured peritoneal macrophages from ScCr and B10 mice were treated with PSP (25 μg/ml) or LPS (100 ng/ml) from *Escherichia coli* O55:B5 (L6529, sigma, USA) for an indicated time period. The supernatants were harvested for detection of cytokine levels and the cell lysates were collected for Western blotting analysis.

For in vivo experiments, ScCr and B10 mice were used to establish the EAC tumor-bearing model. ScCr and B10 tumor-bearing mice were randomly assigned into eight groups. Mice in ScCr saline group and B10 saline group were orally administered with saline for 25 days. Mice in ScCr Adriamycinc (ADM) group and B10 ADM group were treated with 5.33 mg/kg/d ADM (Actavis Italy S.p.A, ITA) through peritoneal injection for the first 3 days, followed by 22 days of saline administration. Mice in ScCr PSP group and B10 PSP group were given PSP (500 mg/kg/d) by oral route for 25 days. Mice in ScCr LPS group and B10 LPS group were orally administered with saline for 25 days and then intraperitoneally injected with LPS (5 mg/kg) 4 h before sacrificed [37]. Twenty-five days after administration, mice were killed by cervical dislocation. The whole body, tumor, spleen and thymus were weighed immediately to calculate the tumor inhibition rate and the immune organ index. The spleen tissue samples were extracted, immediately frozen in liquid nitrogen and then stored at −80 °C for Q-PCR and Western blot analyses.

### Cytokine analysis

Mouse peritoneal macrophages from ScCr and B10 mice were plated in 24-well plates at a density of 2.5 × 10^5^ cells/well and treated with or without PSP (25 μg/ml) or LPS (100 ng/ml) as a positive control. Cells were incubated for 24 h at 37 °C in 5 % CO_2._ Culture supernatants were then collected for determining cytokine concentrations using Mouse TNF-α and IL-6 ELISA kits (4abio, China) according to the manufacturer^’^s instructions.

### Tumor inhibition rate and the immune organ index

The tumor inhibition rate and the immune organ index were calculated as described previously [13]. Briefly, the inhibition rates of growth of EAC solid tumor were calculated according to the formula: inhibition rate (*%*) = (1- mean weight of tumor in administration groups/ mean weight of tumor in the saline group) × 100 *%*. The organ indexes of spleen and thymus were calculated according to the formula: organ index (*%*) = mean weight of organ / body weight × 100 *%*.

### Quantitative real-time PCR assay

Total RNA in spleen homogenates was extracted via RNAiso Plus (Takara, Japan) and reversed transcribed into cDNA with PrimeScript^TM^ RT reagent Kit with gDNA Eraser (Takara, Japan) according to the manufacturer^’^s instructions. Q-PCR was performed on Bio-Rad CFX-96 (Bio-Rad, Foster City, CA, USA), using SYBR® Premix Ex Taq^TM^ II (Takara, Japan). GAPDH and β-actin served as endogenous normalization control (reference genes). The relative expression of mRNA was calculated by Vandesompele Method. The sequences and related information of the Q-PCR primers are shown in Table 1. Amplification began with initial denaturation for 30 s at 95 °C followed by 40 cycles of denaturation at 95 °C for 5 s, annealing and extension at 58 °C for 30 s and then the plate was read. To analyze the specificity of the products, a melt curve procedure was added on Bio-Rad CFX96. Triplicate reactions were run per sample.

**Table 1:** Sequence and useful information of primers designed for detection of mRNA

Gene symbol	Genbank accession no.	Primer sequence (5^’^ → 3^’^)	Product size (bp)	Annealing temperature	E (%)	R^2^
TLR4	NM_021297.2	F ctgggtgagaaatgagctggtaa	122	58 °C	96.5	0.999
		R agccttcctggatgatgttgg				
TRAF6	NM_009424	F catcttcagttaccgacagctcag	131	58 °C	95.2	0.999
		R tggtcgagaattgtaaggcgtat				
NF-κB	NM_008689.2	F ccaaagaaggacacgacagaatc	127	58 °C	95.5	0.999
		R ggcaggctattgctcatcaca				
AP-1	NM_010591.2	F gccctggctgaactgcatag	180	58 °C	91.4	0.998
		R gaagttgctgaggttggcgta				
GAPDH	NM_008084.2	F gacatcaagaaggtggtgaagc	117	58 °C	96.2	0.999
		R gaaggtggaagagtgggagtt				
β-Actin	NM_007393.3	F agattactgctctggctcctagc	147	58 °C	93.6	0.998
		R actcatcgtactcctgcttgct				

*F* Forward primer, *R* Reverse primer, *E* Amplification efficiency, *R* ^*2*^ Correlation coefficient

### Western blot analysis

Mouse peritoneal macrophages and spleen tissues were prepared for Western blot analysis. Protein concentrations were measured by BCA assay (Beyotime, Jiangsu, China), and were separated SDS-PAGE gels and transferred onto PVDF membranes (Millipore, USA). The membranes were blocked and incubated sequentially overnight at 4 °C with antibodies and β-actin.

### Statistical analysis

All values were expressed as means ± standard deviation (SD). Statistical differences between the experimental groups were examined by ANOVA followed by and analyzed by post hoc Student’s Newman-Keuls test with SPSS software (SPSS 18.0). Differences with *P* values of <0.05 were considered significant.

## Abbreviations

PSP: Polysaccharopeptide
TLR4: Toll-like receptors 4
PBMCs: Peripheral blood mononuclear cells
EAC: Ehrlich’sascites carcinoma
ADM: Adriamycin
GLPS: Ganoderma lucidum polysaccharides
IRF5: Interferon regulatory factor 5
NF-κB: Nuclear factor-κB
TRAF6: TNF receptor-associated factor 6
ERK: Extracellular signal-regulated kinase
P38: p38 MAP kinase
JNK: c-Jun N-terminal kinase
AP-1: Activator protein-1
LRR: Leucine rich repeats
PAMPs: Pathogen-associated molecular patterns
TIR: Toll/IL-1 receptor
LPS: Lipopolysaccharide
MyD88: Myeloid differentiation factor 88
TRIF: TIR domain-containing adaptor inducing IFN-β
MAPK: Mitogen-activated protein kinase
